# Migration of carbon nanotubes from liquid phase to vapor phase in the refrigerant-based nanofluid pool boiling

**DOI:** 10.1186/1556-276X-6-219

**Published:** 2011-03-14

**Authors:** Hao Peng, Guoliang Ding, Haitao Hu

**Affiliations:** 1 Institute of Refrigeration and Cryogenics, Shanghai Jiaotong University, 800 Dongchuan Road, Shanghai 200240, China; 2 Key Laboratory of Microgravity (National Microgravity Laboratory)/CAS; Institute of Mechanics, Chinese Academy of Sciences (CAS), 15 Beisihuan Xilu, Beijing 100190, China

## Abstract

The migration characteristics of carbon nanotubes from liquid phase to vapor phase in the refrigerant-based nanofluid pool boiling were investigated experimentally. Four types of carbon nanotubes with the outside diameters from 15 to 80 nm and the lengths from 1.5 to 10 μm were used in the experiments. The refrigerants include R113, R141b and *n*-pentane. The oil concentration is from 0 to 10 wt.%, the heat flux is from 10 to 100 kW·m^-2^, and the initial liquid-level height is from 1.3 to 3.4 cm. The experimental results indicate that the migration ratio of carbon nanotube increases with the increase of the outside diameter or the length of carbon nanotube. For the fixed type of carbon nanotube, the migration ratio decreases with the increase of the oil concentration or the heat flux, and increases with the increase of the initial liquid-level height. The migration ratio of carbon nanotube increases with the decrease of dynamic viscosity of refrigerant or the increase of liquid phase density of refrigerant. A model for predicting the migration ratio of carbon nanotubes in the refrigerant-based nanofluid pool boiling is proposed, and the predictions agree with 92% of the experimental data within a deviation of ±20%.

## Introduction

Nowadays, the researchers show great interest in the possible application of refrigerant-based nanofluids (i.e., the mixtures of nanopowders and conventional pure refrigerants) for improving the performance of refrigeration systems. The researches showed that the refrigerant-based nanofluids have higher thermal conductivity than those of conventional pure refrigerants [1], the addition of nanoparticles enhances the solubility of mineral oil in HFC refrigerant [2], and the addition of nanoparticles can save energy consumption of air-conditioner and refrigerator [3,4]. Comparing with the spherical metal or metal oxide nanoparticles used in these researches, carbon nanotubes (CNTs) have one or two orders of magnitude higher in thermal conductivity, and CNTs can significantly enhance the thermal conductivity of base fluid [5-8] as well as the convective heat transfer coefficient of base fluid [9], so CNTs have great potential for improving the performance of refrigeration systems. For applying CNTs in refrigeration systems, the phase-change heat transfer characteristics of refrigerant-CNT nanofluid and the cycle behavior of CNTs in refrigeration systems should be known. The migration of CNTs from liquid phase to vapor phase in the pool boiling process of refrigerant-CNT nanofluid determines the distribution of CNTs concentration in the liquid phase and vapor phase, and then has significant effect on the phase-change heat transfer characteristics of refrigerant-CNT nanofluid as well as the cycle behavior of CNTs. Therefore, in order to evaluate the phase-change heat transfer characteristics of refrigerant-CNT nanofluid and the cycle behavior of CNTs, the migration of CNTs in the pool boiling process of refrigerant-CNT nanofluid should be researched.

The migration of CNTs from liquid phase to vapor phase in the refrigerant-based nanofluid pool boiling can be divided to the following four physical processes: (1) the departure of bubble from the heating surface, (2) the movement of bubble and CNTs in the liquid phase, (3) the capture of CNTs by bubble, and (4) the escape of CNTs from the liquid-vapor interface. From the above analysis, it can be seen that the interaction between CNTs and bubble is the key factor causing the migration of CNTs from liquid phase to vapor phase. The existing flotation theory can accurately describe the interaction between particles and bubbles. However, they can not directly used to predict the migration of CNTs from liquid phase to vapor phase during pool boiling because they are aimed at the conditions without phase change. The present investigation is beneficial to reveal the mass transfer mechanism of nano-scale solid powders from liquid phase to vapor phase during the phase-change process of fluid, and provides the theoretical basis for evaluating the phase-change heat transfer characteristics of refrigerant-CNT nanofluid and the cycle behavior of CNTs.

Until now, there is no published research on the migration characteristics of CNTs in the pool boiling of refrigerant-based nanofluid. The migration characteristics of nanopowders are mentioned only by the paper of Ding et al. [10], and are focused on one type of spherical nanoparticle (CuO). In the paper, the authors found that the migrated mass of CuO nanoparticles in the pool boiling increases with the increase of the original mass of nanoparticles or the original mass of refrigerant. As the structure and the thermophysical properties of CNTs are different from those of spherical nanoparticles, the migration characteristics of CNTs in the pool boiling of refrigerant-based nanofluid may be different from those of spherical nanoparticles, and should be investigated.

The existing researches on the pool boiling heat transfer characteristics of nanofluids containing CNTs can be divided into three categories as follows: (1) Pool boiling heat transfer of water-CNTs nanofluids. The experimental results showed that CNTs can enhance the pool boiling heat transfer of water [11-13] or deteriorate the pool boiling heat transfer of water [14], and the influence of CNTs on the pool boiling is related to CNTs concentration [13]. (2) Pool boiling heat transfer of pure refrigerant-CNTs nanofluids. The experiments by Park and Jung [11,15] showed that CNTs can enhance the pool boiling heat transfer of pure refrigerants (R22, R123, and R134a), and the enhancement is related to the heat flux. (3) Pool boiling heat transfer of refrigerant-oil mixtures with CNTs. Experiments by Peng et al. [16] showed that CNTs can enhance the pool boiling heat transfer of refrigerant-oil mixtures, and the enhancement increases with the decrease of CNTs' outside diameter or CNTs' nanolubricant mass fraction, while increases with the increase of CNTs' length or CNTs' mass fraction in the CNTs' nanolubricant. From the above researches, it can be seen that the CNTs physical dimension (i.e., the outside diameter and the length of CNTs), refrigerant type, CNTs concentration, oil concentration, and heat flux have influences on the pool boiling heat transfer of refrigerant-based nanofluid. Therefore, the influences of the above factors on the migration characteristics of CNTs need be concerned. In addition, the initial liquid-level height affects the pool boiling heat transfer, so the influence of initial liquid-level height on the migration characteristics of CNTs also needs be concerned.

The objective of this paper is to experimentally investigate the influences of CNTs physical dimension, refrigerant type, oil concentration, heat flux, and initial liquid-level height on the migration characteristics of CNTs in the refrigerant-based nanofluid pool boiling at different original CNTs concentrations, and to propose a model for predicting the migration ratio of CNTs in the refrigerant-based nanofluid pool boiling.

## Experiments

### Test conditions and experimental objects

Test conditions are divided into five categories, as tabulated in Table 1.

**Table 1:** Test conditions

Objective of investigation	CNTs type	Refrigerant type	Oil concentration *x*_o _(wt.%)	Heat flux *q *(kWm^-2^)	Initial liquid-level height *L *(cm)	Original CNTs concentration *φ*_n _(vol.%)
Influence of CNTs physical dimension on migration	CNT#1, CNT#2, CNT#3, CNT#4	R113	0	50	2.0	0.56, 1.11, 1.65, 2.19, 2.72, 3.25, 3.77
Influence of refrigerant type on migration	CNT#2	R113, R141b, *n*-pentane	0	50	2.0	0.56, 1.11, 1.65, 2.19, 2.72, 3.25, 3.77
Influence of oil concentration on migration	CNT#2	R113	1, 5, 10	50	2.0	0.56, 1.11, 1.65, 2.19, 2.72, 3.25, 3.77
Influence of heat flux on migration	CNT#2	R113	0	10, 20, 50, 100	2.0	0.56, 1.11, 1.65, 2.19, 2.72, 3.25, 3.77
Influence of initial liquid-level height on migration	CNT#2	R113	0	50	1.3, 2.0 2.7, 3.4	0.56, 1.11, 1.65, 2.19, 2.72, 3.25, 3.77

**Figure 1:**
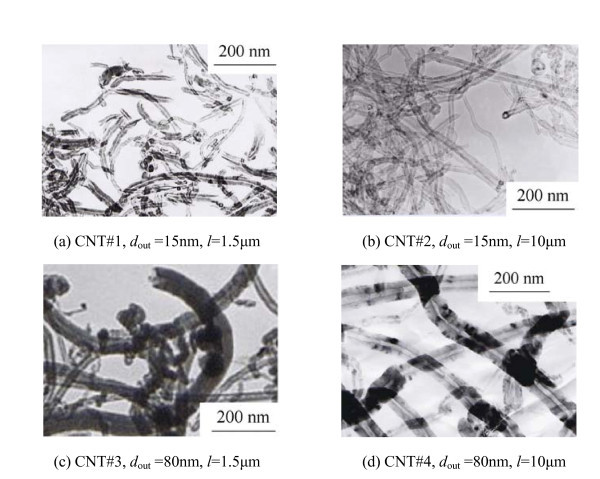
**TEM photographs of CNTs**. **(a)** CNT#1;** (b)** CNT#2; **(c) **CNT#3; **(d)** CNT#4.

The objective of category 1 is to investigate the influences of CNTs physical dimension on the migration characteristics of CNTs. Four types of CNTs with different physical dimensions (numbered as CNT#1, CNT#2, CNT#3, and CNT#4) produced by the chemical vapor deposition method are used in these test conditions. The physical dimensions of these four types of CNTs are shown in Table 2 and the TEM (transmission electron microscope) photographs of the CNTs are shown in Figure 1. In these test conditions, the other influence factors including the refrigerant type, oil concentration, heat flux and initial liquid-level height are fixed.

**Table 2:** Physical dimensions of CNTs

Property	Mean outside diameter (*d*_out_)	Mean inside diameter (*d*_in_)	Mean length (*l*)	Aspect ratio (*l*/*d*_out _)
CNT#1	15 nm	10 nm	1.5 μm	100
CNT#2	15 nm	10 nm	10 μm	666.7
CNT#3	80 nm	18 nm	1.5 μm	18.8
CNT#4	80 nm	18 nm	10 μm	125

The objective of category 2 is to investigate the influences of refrigerant type on the migration characteristics of CNTs. Three types of refrigerants including R113, R141b, and *n*-pentane are used in these test conditions, belonging to CFC refrigerant, HCFC refrigerant, and alkane refrigerant, respectively. The reasons for choosing these three types of refrigerants are as follows: (1) R113, R141b and *n*-pentane are in liquid state at room temperature and atmospheric pressure while the widely used refrigerants (e.g., R410A) are in vapor state, so it is much easier to prepare refrigerant-based nanofluids based on R113, R141b, or *n*-pentane. (2) These three types of refrigerants have different chemical and thermophysical properties including molecular mass, density, dynamic viscosity, etc. The properties of these three refrigerants are given in Table 3. In these test conditions, the other influence factors including the CNTs physical dimension, oil concentration, heat flux and initial liquid-level height are fixed.

**Table 3:** Properties of refrigerants in the experiments

Property	R113	R141b	*n*-Pentane
Chemical formula	Cl2FC-CClF2	CH3CCl2F	C5H12
Molecular mass (g/mol)	187.37	116.95	72.15
Normal boiling point (^°^C)	47.6	32.05	36.1
Liquid-phase density (kg·m^-3^)	1508	1220	606
Vapor-phase density(kg·m^-3^)	7.4	4.8	3.2
Liquid-phase dynamic viscosity (Pa·s)	5 × 10^-4^	3.78 × 10^-4^	1.91 × 10^-4^
Liquid-phase isobaric specific heat (J·kg^-1^·K^-1 ^)	940	1,165	2,441
Surface tension (N·m^-1^)	0.0147	0.0173	0.0138

The objective of category 3 is to investigate the influences of oil concentration on the migration characteristics of CNTs. The lubricating oil RB68EP is used in the experiments. RB68EP is an ester oil with a density of 0.964 g·cm^-3 ^at 15°C and kinematic viscosities of 66.79 and 8.23 mm^2·^s^-1 ^at 40°C and 100°C, respectively, as reported by the manufacturer. The oil concentration is from 0 to 10 wt.%, covering the oil concentration in the actual refrigeration system. In these test conditions, the other influence factors including the CNTs physical dimension, refrigerant type, heat flux and initial liquid-level height are fixed.

The objective of category 4 is to investigate the influences of heat flux on the migration characteristics of CNTs. The heat flux is from 10 to 100 kW·m^-2^, covering the heat flux in the actual refrigeration system. In these test conditions, the other influence factors including the CNTs physical dimension, refrigerant type, oil concentration and initial liquid-level height are fixed.

The objective of category 5 is to investigate the influences of initial liquid-level height on the migration characteristics of CNTs. The initial liquid-level height is from 1.3 to 3.4 cm. In these test conditions, the other influence factors including the CNTs physical dimension, refrigerant type, oil concentration, and heat flux are fixed.

### Experimental apparatus

**Figure 2:**
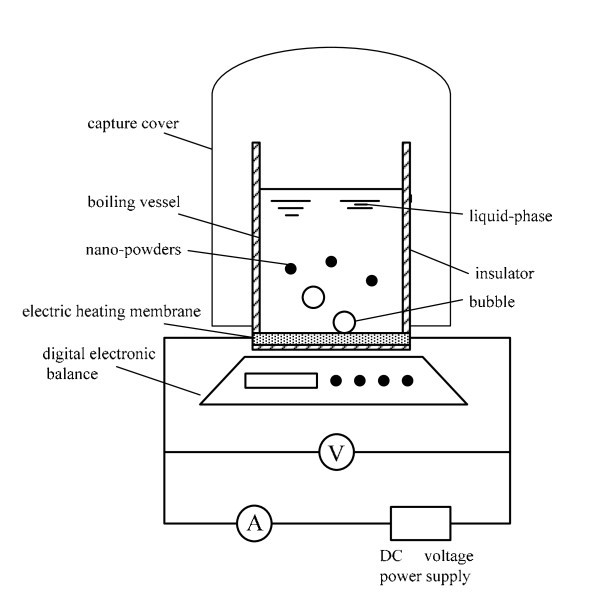
**Schematic diagram of experimental apparatus**.

The experimental apparatus used for testing the migration characteristics of CNTs in the refrigerant-based nanofluid pool boiling mainly consists of a pool boiling device, a capture cover and a digital electronic balance, as schematically shown in Figure 2. The pool boiling device mainly consists of a boiling vessel and an electric heating membrane. The boiling vessel is a cylindrical glass container with the inside diameter of 50 mm and the height of 95 mm. The vessel is insulated with glass fibers to reduce heat loss to the surroundings. The electric heating membrane is connected with the direct-current voltage power supply. The ampere meter with the calibrated precision of 0.5% is used for reading electric current supplied to the heating surface, and a data acquisition system with the calibrated precision of 0.002% is used to measure the voltage across the heating surface. The heat flux through the heating surface is controlled by adjusting the heating power of the plate heater, and is calculated by the measured electric current, voltage, and heating surface area. The uncertainty of heat flux is estimated to be smaller than 1.2%. The capture cover is used to collect the CNTs spouted to the environment. The measurement range of the digital electronic balance is from 10.0 mg to 210.0000 g, and the maximum error is 0.1 mg. All the experiments are performed at atmospheric pressure (101.3 kPa) by venting the boiling vessel to ambient.

### Experimental method

The objective of the measurements is to get the migrated mass of CNTs from liquid phase to vapor phase in the refrigerant-based nanofluid pool boiling. Ding et al. [10] have proposed the weighing method to obtain the migrated mass of spherical nanoparticles, and this method is also used in the present study to get the migrated mass of CNTs.

The experimental procedure for the refrigerant-based nanofluid without oil consists of the following steps: (1) adding CNTs with the original mass of *m*_n0 _to the boiling vessel; (2) weighing the total mass of the boiling vessel and the CNTs, *m*_1_; (3) adding refrigerant with the original mass of *m*_r0 _to the boiling vessel; (4) opening the direct-current voltage power supply and heating the refrigerant-based nanofluid to be boiling; (5) adjusting the voltage to control the heat flux; (6) weighing the total mass of the boiling vessel and the CNTs, *m*_2_, when the refrigerant is entirely evaporated (the signal for the entire evaporation is that the mass of mixture does not change for 12 h); (7) calculating the migrated mass of CNTs with the equation Δ*m*_n _= *m*_1 _- *m*_2_.

The experimental procedure for the refrigerant-based nanofluid with oil consists of the following steps: (1) adding CNTs with the original mass of *m*_n0 _and oil with the mass of *m*_o _to the boiling vessel; (2) weighing the total mass of the boiling vessel, the CNTs and oil, *m*_3_; (3) adding refrigerant with the original mass of *m*_r0 _to the boiling vessel; (4) opening the direct-current voltage power supply and heating the refrigerant-based nanofluid with lubricating oil to be boiling; (5) adjusting the voltage to control the heat flux; (6) weighing the total mass of the boiling vessel, the CNTs and oil, *m*_4_, when the refrigerant is entirely evaporated (the signal for the entire evaporation is that the mass of mixture does not change for 12 h); (7) calculating the migrated mass of CNTs with the equation Δ*m*_n _= *m*_3 _- *m*_4_.

### Data reduction and uncertainty

In order to quantitatively evaluate the migration degree of CNTs, the migration ratio of CNTs, *ζ*, is defined by Eq. 1:(1)

where, Δ*m*_n _is the migrated mass of CNTs, and *m*_n0 _is the original mass of CNTs.

The original CNTs concentration is defined as the original volume fraction of CNTs in the liquid phase (liquid refrigerant or liquid refrigerant-oil mixture), presented as Eq. 2:(2)

where, *m*_r0_, *m*_o _are the original mass of refrigerant and the mass of oil, respectively; *ρ*_n_, *ρ*_r, L_, *ρ*_o _are the density of CNTs, liquid-phase refrigerant and oil, respectively.

The oil concentration is defined as the original mass fraction of oil in the liquid refrigerant-oil mixture, presented as Eq. 3:(3)

The relative uncertainty of migration ratio of CNTs is calculated as:(4)

Determined by the accuracy of the digital electronic balance, the maximum uncertainties of the measured migrated mass of CNTs (*δ*Δ*m*_n_) and original mass of CNTs (*δm*_0_) are 0.2 mg and 0.1 mg, respectively. The maximum relative uncertainty of migration ratio of CNTs is obtained at the condition of the smallest migrated mass of CNTs and the migrated mass of CNTs, and calculated to be 2.5%.

Tests under several conditions were repeated for three times, and it shows that the differences among the three testing results under each condition are less than 3%. Therefore, the experimental results are reproducible.

## Experimental results and analysis

### Influence of CNTs physical dimension on the migration of CNTs

**Figure 3:**
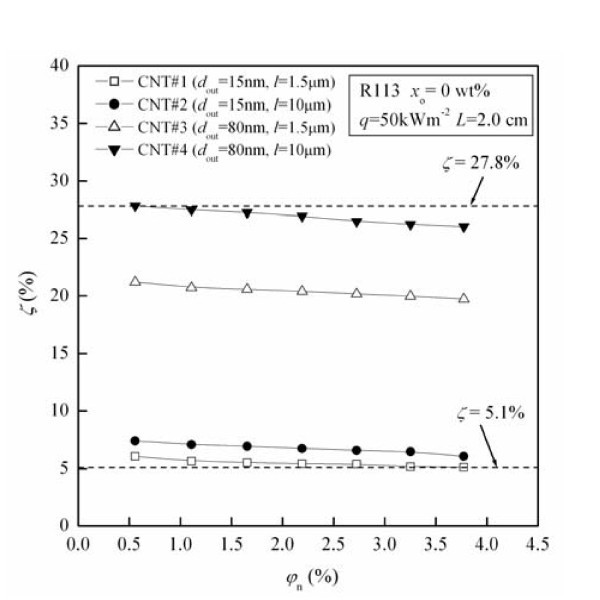
**Influence of CNTs physical dimension on the migration ratio of CNTs**.

Figure 3 shows the migration ratio (*ζ*) of CNTs as a function of original CNTs concentration (*φ*_n_) for CNTs with different physical dimensions. The values of *ζ *under these test conditions are in the range of 5.1% to approximately 27.8%. For fixed CNTs physical dimension, *ζ *decreases with the increase of *φ*_n_. For example, *ζ *for CNT#1 decreases by 15.8% with the increase of *φ*_n _from 0.56 to 3.77 vol.%.

From Figure 3 it can be seen that the migration ratio (*ζ*) of CNTs increases with the increase of the outside diameter of CNTs (*d*_out_) when the length of CNTs (*l*) is fixed. For example, at the condition of *l *= 1.5 μm, the value of *ζ *increases by maximally 286.4% with the increase of *d*_out _from 15 to 80 nm. It also can be seen from Figure 3 that the migration ratio (*ζ*) of CNTs increases with the increase of the length of CNTs (*l*) when the outside diameter of CNTs (*d*_out_) is fixed. For example, at the condition of *d*_out _= 15 nm, the value of *ζ *increases by maximally 25.7% with the increase of *l *from 1.5 to 10 μm. The possible reasons for the above phenomenon are as follows: The capture of CNTs by the bubbles generated in the pool boiling leads to the migration of CNTs. Brownian diffusion, interception, gravity settling and inertial impaction are four mechanisms for the capture of particles by bubbles [17]. As the CNTs do not exhibit Brownian motion due to their high aspect ratio [18,19], the capture efficiency of CNTs by bubbles can be considered as the sum of the capture efficiencies caused by interception, gravity settling, and inertial impaction. Each of the above three captured efficiencies increases with Stokes diameter of CNTs. The increase of the outside diameter or the length of CNTs causes the increase of Stokes diameter of CNTs [20], thus the captured efficiency of CNTs by bubbles increases, which leads to the migration ratio (*ζ*) of CNTs increasing with increase of the outside diameter or the length of CNTs.

### Influence of refrigerant type on the migration of CNTs

**Figure 4:**
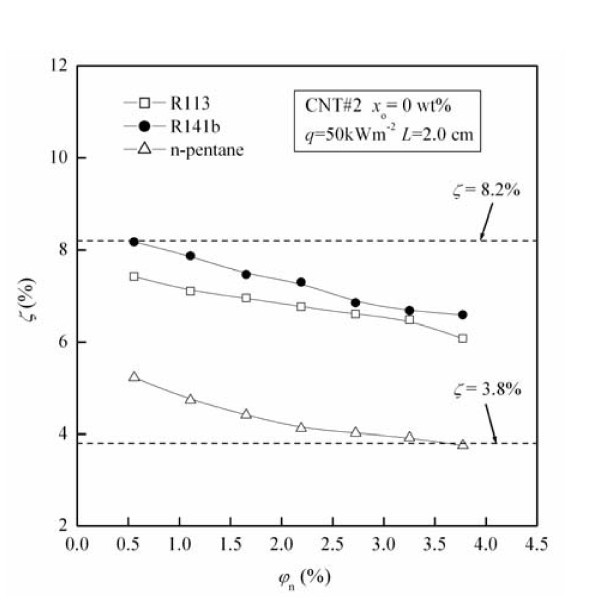
**Influence of refrigerant type on the migration ratio of CNTs**.

Figure 4 shows the migration ratio (*ζ*) of CNTs as a function of original CNTs concentration (*φ*_n_) for different types of refrigerants. The values of *ζ *under these test conditions are in the range of 3.8% to approximately 8.2%. For fixed refrigerant type, *ζ *decreases with the increase of *φ*_n_. For example, *ζ *for R141b decreases by 19.5% with the increase of *φ*_n _from 0.56 to 3.77 vol.%.

From Figure 4 it can be seen that the migration ratio (*ζ*) of CNTs are in the order of R141b > R113 >*n*-pentane, the value of *ζ *in the R141b-based nanofluid is by maximally 10.7% larger than that in the R113-based nanofluid, and is by maximally 77.4% larger than that in *n*-pentane-based nanofluid. The possible reasons for the above phenomenon are as follows: (1) The dynamic viscosity values for these three refrigerants are in the order of R113 (5 × 10^-4 ^Pa·s) > R141b (3.78 × 10^-4 ^Pa·s) >*n*-pentane (1.91 × 10^-4 ^Pa·s). The larger dynamic viscosity causes the smaller capture efficiencies caused by gravity settling and inertial impaction, which leads to the smaller migration ratio of CNTs. (2) The liquid-phase density values of for these three refrigerants are in the order of R113 (1,508 kg·m^-3^) > R141b (1,220 kg·m^-3^) >*n*-pentane (606 kg·m^-3^). The larger liquid-phase density means the larger mass of liquid-phase refrigerant at fixed liquid-level height, thus the amount of bubbles generated in the pool boiling is larger, which leads to the larger migration ratio of CNTs. The influence of refrigerant type on the migration ratio of CNTs is determined by the conjunct role of the above two aspects, and follows the order of R141b > R113 >*n*-pentane. It can be concluded that the migration ratio of carbon nanotube increases with the decrease of dynamic viscosity of refrigerant or the increase of liquid-phase density of refrigerant.

### Influence of oil concentration on the migration of CNTs

**Figure 5:**
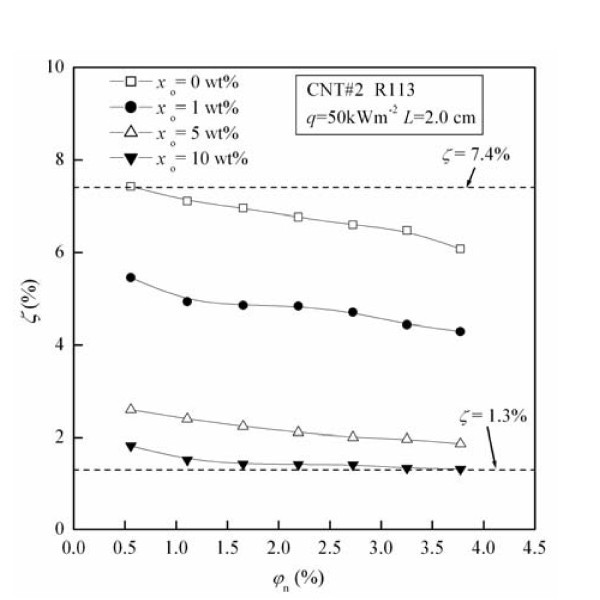
**Influence of oil concentration on the migration ratio of CNTs**.

Figure 5 shows the migration ratio (*ζ*) of CNTs as a function of original CNTs concentration (*φ*_n_) for different oil concentrations (*x*_o_). The values of *ζ *under these test conditions are in the range of 1.3% to approximately 7.4%. For fixed *x*_o_, *ζ *decreases with the increase of *φ*_n_. For example, at the condition of *x*_o _= 5 wt.%, *ζ *decreases by 28.2% with the increase of *φ*_n _from 0.56 to 3.77 vol.%.

From Figure 5 it can be seen that the migration ratio (*ζ*) of CNTs decreases by maximally 70.7% with the increase of oil concentration (*x*_o_) from 1 to 10 wt.%. The possible reasons are as follows: (1) The dynamic viscosity and surface tension of lubricating oil RB68EP are larger than those of pure refrigerant, causing the dynamic viscosity and surface tension of liquid-phase refrigerant-oil mixture increase with the increase of *x*_o_. (2) The increase of dynamic viscosity of liquid-phase refrigerant-oil mixture results in the decrease of capture efficiencies caused by gravity settling and inertial impaction, which leads to *ζ *decreasing with the increase of *x*_o_. (3) The increase of surface tension of liquid-phase refrigerant-oil mixture causes the increase of bubble departure diameter in the pool boiling, thus the capture efficiencies caused by interception, gravity settling, and inertial impaction decrease, which leads to *ζ *decreasing with the increase of *x*_o_.

### Influence of heat flux on the migration of CNTs

**Figure 6:**
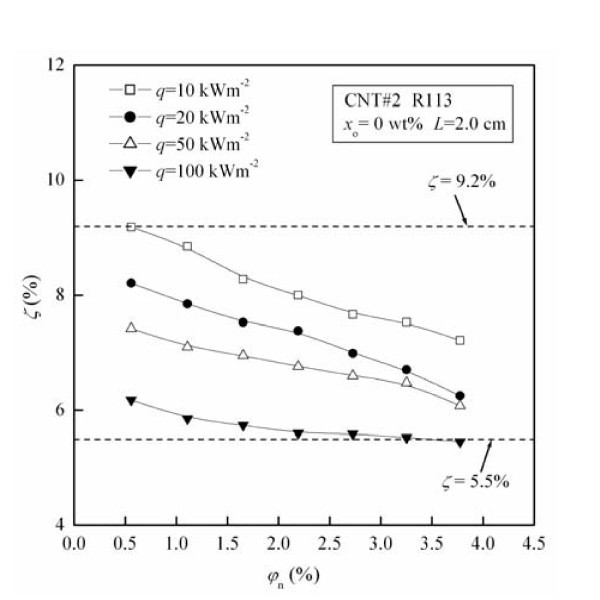
**Influence of heat flux on the migration ratio of CNTs**.

Figure 6 shows the migration ratio (*ζ*) of CNTs as a function of original CNTs concentration (*φ*_n_) for different heat fluxes (*q*). The values of *ζ *under these test conditions are in the range of 5.5% to approximately 9.2%. For fixed *q*, *ζ *decreases with the increase of *φ*_n_. For example, at the condition of *q *= 10 kWm^-2^, *ζ *decreases by 21.5% with the increase of *φ*_n _from 0.56 to 3.77 vol.%.

From Figure 6 it can be seen that the migration ratio (*ζ*) of CNTs decreases by maximally 33.9% with the increase of heat flux (*q*) from 10 to 100 kWm^-2^. The possible reasons are as follows: (1) The increase of heat flux causes the increase of the velocity of departure bubble [21], thus the velocity of rising bubble increases. (2) The increase of velocity of rising bubble results in the decrease of capture efficiency caused by gravity settling, which leads to the decrease of *ζ*. (3) The increase of velocity of rising bubble results in the increase of capture efficiency caused by inertial impaction, which leads to the increase of *ζ*. (4) The increase of velocity of rising bubble results in the decrease of the bubble rising time in the liquid phase, causing the decrease of the amount of CNTs captured by bubbles, which leads to the decrease of *ζ*. The conjunct role of the above aspects leads to the migration ratio of CNTs (*ζ*) decreasing with the increase of heat flux.

### Influence of liquid-level height on the migration of CNTs

**Figure 7:**
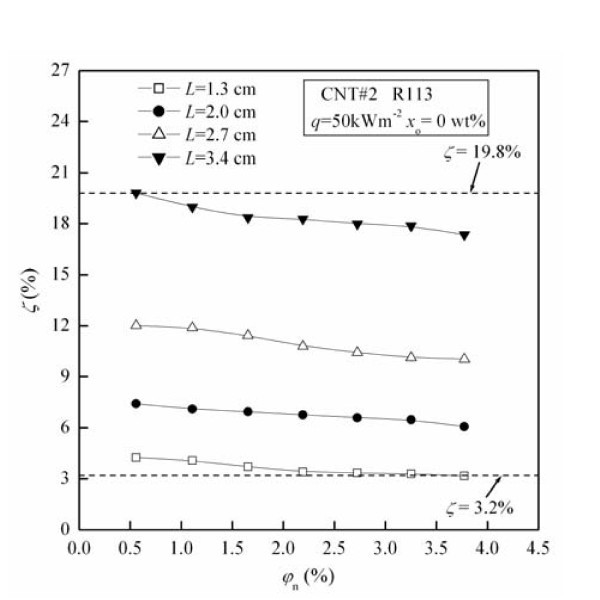
**Influence of initial liquid-level height on the migration ratio of CNTs**.

Figure 7 shows the migration ratio (*ζ*) of CNTs as a function of original CNTs concentration (*φ*_n_) for different initial liquid-level heights (*L*). The values of *ζ *under these test conditions are in the range of 3.2% to approximately 19.8%. For fixed *L*, *ζ *decreases with the increase of *φ*_n_. For example, at the condition of *L *= 2.7 cm, *ζ *decreases by 16.5% with the increase of *φ*_n _from 0.56 to 3.77 vol.%.

From Figure 7 it can be seen that the migration ratio (*ζ*) of CNTs increases by maximally 446.9% with the increase of initial liquid-level height (*L*) from 1.3 to 3.4 cm. The possible reasons are as follows: (1) The increase of initial liquid-level height causes the increase of the bubble rising time in the liquid phase, thus the amount of CNTs captured by bubbles, which leads to *ζ *increasing with the increase of liquid-level height. (2) The increase of initial liquid-level height causes the increase of CNTs escape probability on the liquid-vapor interface, which leads to *ζ *increasing with the increase of liquid-level height.

## Prediction of migration ratio of CNTs in the refrigerant-based nanofluid pool boiling

As there is no published literature on the model for predicting the migration ratio of CNTs in the refrigerant-based nanofluid pool boiling, the development of a new model is needed. The CNTs physical dimension, refrigerant type, oil concentration, heat flux, and initial liquid-level height are five important factors influencing the migration of CNTs, and should be reflected in the new model.

From the beginning of the pool boiling *t*_0 _to the moment of *t*, the migration ratio of CNTs can be expressed as:(5)

where, *m*_n,*t *_is the mass of CNTs in the liquid phase at the moment of *t*; *m*_n0 _is the original mass of CNTs.

According to the principle of mass conservation, the mass of CNTs in the liquid phase changed with time can be expressed as:(6)

where, *K *is the migration coefficient of CNTs (i.e., the migration proportion of CNTs caused by single bubble); *n*_b _is the number of generated bubbles per unit time.

From Eq. 6, the following equation can be obtained.(7)

Combing Eqs. 5 and 7,  can be calculated as:(8)

Therefore, from the beginning to the end of pool boiling, the migration ratio of CNTs is:(9)

In Eq. 9, *N*_b _is the total number of generated bubbles from the beginning to the end of pool boiling.

### 1. The calculation of *K*

The migration of CNTs can be considered as the capture of CNTs by bubbles combining the escape of CNTs from the liquid-vapor interface. In order to describe the capture process of CNTs by bubbles, the capture efficiencies of CNTs caused by interception, gravity settling and inertial impaction should be included in *K*. In order to describe the escape process of CNTs from the liquid-vapor interface, the escape probability of CNTs should be included in *K*. The original CNTs concentration has influences on the bubble diameter and bubble rising velocity during the pool boiling process of refrigerant-based nanofluid, and then has influence on the migration of CNTs. Therefore, the CNTs concentration impact factor should also be included in *K*. The expression of *K *is as follows:(10)

where, *α*_I_, *α*_G_, and *α*_IN _are the capture efficiencies of CNTs caused by interception, gravity settling, and inertial impaction, respectively; *β *is the escape probability of CNTs; *γ *is the CNTs concentration impact factor. *α*_I_, *α*_G_, *α*_IN_, *β*, and *γ *are expressed as Eqs. 11 to 15, respectively.(11)(12)(13)(14)(15)

In Eqs. 11 to 15, *D *and *H *are the bottom diameter and height of boiling vessel, respectively; *ρ*_L_, *ρ*_b_, *ρ*_n _are the density of liquid refrigerant-oil mixture, bubble and CNT, respectively; * μ*_L _is the dynamic viscosity of liquid refrigerant-oil mixture; *d*_s _is the Stokes diameter; *d*_b _is the diameter of bubble; *u*_b _is the bubble rising velocity. The calculation of *ρ*_L _and *m*_L _are presented in -Table 4.

**Table 4:** Calculation of the properties of liquid refrigerant-oil mixture

Property	Model for calculating property	Author
Specific heat (J·kg^-1^·K^-1^)	*C*_*p*,L_ = (1 - *x*_o_)C_*p*,r_ + *x*_o_*C*_*p*,o_(A1)	Jensen and Jackman [23]
Viscosity (Pa·s)	(A2)	Kedzierski and Kaul [24]
Surface tension (N·m^-1^)	*σ *= *σ*_r_ + (*σ*_o_ - *σ*_r_)*x*_o_^0.5^(A3)	Jensen and Jackman [23]
Density (kg·m^-3^)	(A8)	

*d*_s _can be calculated by Eq. 16 [20]:(16)

*d*_b _can be calculated by Cole-Rohsenow correlation [22], as shown in Eq. 17:(17)

*u*_b _can be calculated by Malenkov correlation [21], as shown in Eq. 18:(18)

In Eqs. 17 and 18, *C*_*p*, L_, is the isobaric specific heat of liquid refrigerant-oil mixture; *σ *is the surface tension of refrigerant-oil mixture; *T*_sat _is the saturation temperature; *h*_fg _is the latent heat of vaporization. The calculation of *C*_*p*, L _and *σ *are presented in -Table 4.

### 2. The calculation of *N*_b_

As the original mass of refrigerant is equal to the total mass of generated bubbles from the beginning to the end of pool boiling, *N*_b _can be calculated by Eq. 19.(19)

The nine coefficients of *a*_1_, *a*_2_, *a*_3_, *a*_4_, *b*_1_, *b*_2_, *b*_3_, *b*_4_, and *b*_5 _in Eqs. 11 to 15 are fitted based on total 105 experimental data in this study. By nonlinear programming solution method, the nine coefficients of *a*_1_, *a*_2_, *a*_3_, *a*_4_, *b*_1_, *b*_2_, *b*_3_, *b*_4_, and *b*_5 _can be determined as 0.1, 6.3 × 10^-6^, 1,995.3, 66.9, 2.34, 0.4, 2.26, 8.78, and -0.09, respectively. Therefore, the model for predicting the migration ratio of CNTs in the refrigerant-based nanofluid pool boiling is expressed Eq. 20.(20)

**Figure 8:**
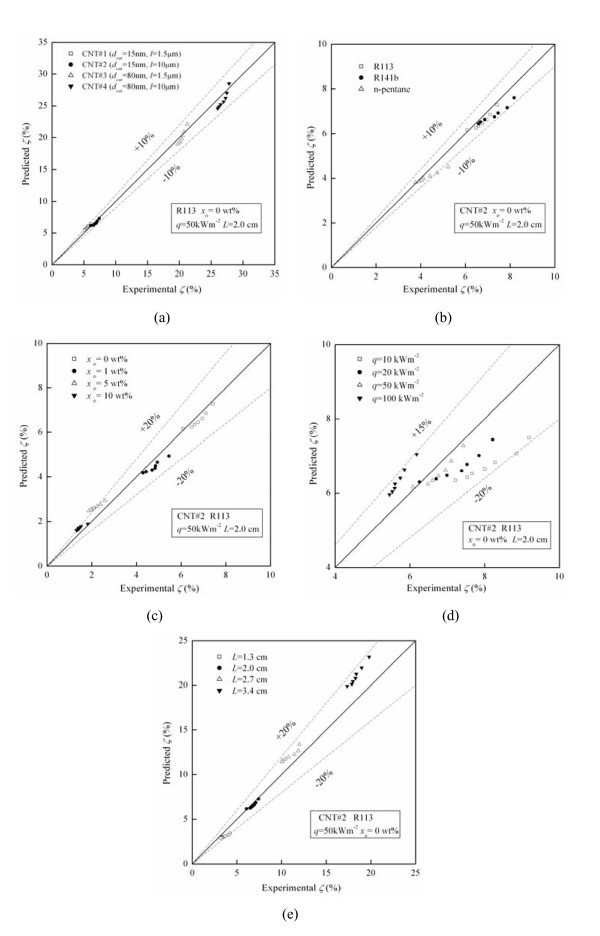
**Comparison between the predicted migration ratios of the model with the experimental data**. **(a)** for different CNTs physical dimensions; **(b) **for different refrigerant types; **(c)** for different oilconcentrations; **(d)** for different heat fluxes; **(e)** for different initial liquid-level heights.

Figure 8a to e shows the comparison between the predicted values of the model with the experimental data for different CNTs physical dimensions, refrigerant types, oil concentrations, heat fluxes, and liquid-level heights, respectively. It can be seen from Figure 8a to e that the migration ratio of CNTs predicted by the model and the experimental data have the same tendency changing with the CNTs physical dimension, refrigerant type, oil concentration, heat flux, or initial liquid-level height. The predicted values of the model agree with 92% of the experimental data of migration ratio of CNTs within a deviation of ± 20%, and the mean deviation is 9.96%.

## Conclusions

Migration characteristics of CNTs from liquid phase to vapor phase in the pool boiling process of refrigerant-based nanofluid are investigated experimentally, and some conclusions are obtained.

1. The migration ratio of CNTs increases with the increase of the outside diameter of CNTs or the length of CNTs.

2. The migration ratio of carbon nanotube increases with the decrease of dynamic viscosity of refrigerant or the increase of liquid-phase density of refrigerant. Under the present experimental conditions, the migration ratio of CNTs in the R141b-based nanofluid is by maximally 10.7% larger than that in the R113-based nanofluid, and is by maximally 77.4% larger than that in *n*-pentane-based nanofluid.

3. The migration ratio of CNTs decreases with the increase of oil concentration. Under the present experimental conditions, the migration ratio decreases by maximally 70.7% with the increase of oil concentration from 1 to 10 wt.%.

4. The migration ratio of CNTs decreases with the increase of heat flux. Under the present experimental conditions, the migration ratio decreases by maximally 33.9% with the increase of heat flux from 10 to 100 kWm^-2^.

5. The migration ratio of CNTs increases with the increase of initial liquid-level height. Under the present experimental conditions, the migration ratio increases by maximally 446.9% with the increase of initial liquid-level height from 1.3 to 3.4 cm.

6. A model for predicting the migration ratio of CNTs in the refrigerant-based nanofluid pool boiling is proposed, and the predicted values of the model can agree with 92% of the experimental data of migration ratio of CNTs within a deviation of ± 20%.

## Competing interests

The authors declare that they have no competing interests.

## Authors' contributions

HP carried out the experimental study and model development. GD participated in the model development and design of the experiments. HH participated in the experimental study. All authors read and approved the final manuscript.
